# Oxidative stress responses and histological changes in the liver of Nile tilapia exposed to silver bulk and nanoparticles

**DOI:** 10.1038/s41598-025-97731-8

**Published:** 2025-05-02

**Authors:** Hala E. Ghannam, Alaa I. Khedr, Radwa El-Sayed, Nasr M. Ahmed, Sally M. Salaah

**Affiliations:** 1 National Institute of Oceanography and Fisheries, NIOF, Cairo, Egypt; 2 Department of Zoology, Faculty of Women for Arts, Science and Education, Ain Shams University, Cairo, 11757 Egypt

**Keywords:** Silver nanoparticles, Ecotoxicology, Antioxidant, Oxidative stress, Histopathological changes, Environmental impact, Freshwater ecology, Marine biology, Enzymes, Animal physiology

## Abstract

The increased utilization of silver nanoparticles (AgNPs) in multiple applications is leading to a rise in environmental contamination caused by their release, particularly in aquatic ecosystems. This study investigates the effects of different concentrations of AgNPs (10, 20, 50, and 100 µg/L) and bulk silver nitrate (AgNO_3_) at 100 µg/L, on the hepatic antioxidant defense system, oxidative stress markers, and liver histopathology of Nile tilapia (Oreochromis niloticus), with sampling conducted biweekly over six weeks. AgNPs were chemically synthesized using trisodium acetate, yielding an average crystallite size of 29.92 nm. Results demonstrated that both antioxidant enzyme activities and lipid peroxidation (LPO) levels in Nile tilapia exhibited a dose-dependent response. During weeks 2 and 4, superoxide dismutase (SOD), catalase (CAT), glutathione peroxidase (GPx), glutathione reductase (GR), activities, along with LPO levels were significantly increased, while TAC levels notably decreased, especially at higher AgNPs concentrations. By week 6, antioxidant enzyme activities were suppressed, and LPO levels were markedly elevated in the higher AgNPs groups (AgNPs-50 and AgNPs-100). In contrast, fish exposed to bulk AgNO_3_ exhibited activation of the enzymatic antioxidant system, although LPO levels remained elevated throughout the experimental period. Histopathological analysis revealed progressive liver damage, including congestion, dilation, fibrosis, fatty degeneration, and necrosis. These effects were more pronounced with higher doses of AgNPs. The results showed a mitigation response among all experimental groups during the first four weeks. However, by week 6, the antioxidant system in Nile tilapia exposed to higher doses of AgNPs failed to cope with the induced oxidative stress. This underscores the significantly higher ecological risks associated with prolonged exposure to AgNPs compared to AgNO_3_, revealing a critical concern for the stability and health of aquatic ecosystems.

## Introduction

Nanotechnology has drawn considerable interest due to its extensive use across multiple sectors, such as medicine, environmental science, agriculture, and industry^1^. The use of synthetic nanomaterials is growing due to their vast applications in many fields such as biology, pharmaceuticals, biomedicine, textiles, cosmetics, healthcare technology, home appliances, and electronics^2^. However, the accumulation of these nanomaterials in freshwater environments, primarily from anthropogenic activities, is leading to denaturation of these ecosystems^3,4^. Considering their extensive applications, the effect of nanomaterial pollution on aquatic organisms is a main consideration, as these substances are extremely harmful, persistent, durable, and have substantial bioaccumulative tendency^5^.

In aquatic ecosystems, evaluating the bioavailability and perilous nature of nanoparticles (NPs) to aquatic organisms, can be an effective tool to assess the impact of NPs. Nanoparticles can interact with their ionic forms and amplify their concentrations, which may have adverse effects on aquatic life^6–9^. Silver is widely used in aquaculture owing to its potent antimicrobial and biocidal characteristics^10^. Moreover, Silver nanoparticles (AgNPs) are among the most prevalent synthesized nanomaterials, due to their exceptional catalytic activity to combined with other materials, as well as its thermal and conductive efficiency and affordable production^11,12^. Among AgNPs synthetization techniques, the chemical reduction is the most common one, as it is a suitable, more versatile method to produce large amount of AgNPs at a low cost^13^.

Silver can disrupt many vital processes in fish, injuring organs membranes, inducing body disfigurements, oxidative stress, disrupting gene expression, and other consequences^14,15^. Hence, the discharge of sliver into aquatic ecosystems even at small amounts (in ng) can pose a substantial and persistent risks. Both concentration and particle size are significant factors affecting the toxicity of AgNPs, as they directly impact their dissolution, surface charge, and aggregation^16^.

Moreover, the environmental repercussions of NPs accumulation are generally unidentified, especially for freshwater ecosystems and marine environments. Due to their nanoscale size, these NPs can simply enter the cell by passing the cell membranes, causing serious cellular damages across various cell types^17^.

Aquatic organisms are extensively used in ecotoxicology assessments due to their prevalent occurrence, especially fish. Fish are excellent bioindicators for assessing environmental contaminates due to their sensitivity to aquatic pollutants^18^. Nile tilapia (*O. niloticus*) is a native fish to Africa, especially Egypt, yet, there is limited research on the toxic mechanisms of AgNPs compared to AgNO_3_ and their physiological effects in fish^15^. While toxic metals such as copper or cadmium are extensively investigated, there is limited number of comprehensive studies on AgNPs versus AgNO_3_ toxicity in aquatic environments^19^. Given the insufficient research on the toxic effects of AgNPs and their ionic form (AgNO_3_) on aquatic organisms, the present study aims to provide a comparative toxicological analysis of synthesized AgNPs and AgNO_3_. This study was designed to assess and compare the effects of prolonged exposure (six weeks) to AgNPs and AgNO_3_ on hepatic antioxidant enzymes, oxidative stress markers, and histopathological alterations in Nile tilapia (*O. niloticus*).

## Materials and methods

### Chemicals

Silver nitrate (AgNO_3_), Trisodium acetate pentahydrate (C_6_H_7_Na_3_O_8_) and Sodium hydroxide (NaOH) were obtained from sigma Aldrich.

### Synthesis of AgNPs

Silver nanoparticles (AgNPs) were manufactured using the chemical reduction method outlined by^20^, with some adjustments made. Trisodium acetate was applied as a stabilizing and reducing agent. 250 mL of 1 mM silver nitrate solution (AgNO_3_) was prepared in deionized water at 90 ⁰C for 1 h. 100 mL of trisodium acetate solution was added dropwise to the aqueous AgNO_3_ solution with stirring at 90 ⁰C, while the pH of the solution medium was adjusted to 9 using NaOH solution. The solution turned dark brown upon the addition of trisodium acetate solution, which confirms the reduction of silver ions^21^. A complete precipitation of the formed AgNPs occurred at pH 12. The solution was stirred for 2 h, and then aged for 1 day. A high-speed centrifuge was used to separate the precipitate at 10,000 rpm. The precipitate was then washed several times with double distilled water until a neutral pH was achieved. Finally, it was dried in an oven at 60 ⁰C then ground in a mortar to obtain fine particles.

### AgNPs characterization methods

AgNPs powder samples (0.5 g) were utilized for XRD analysis using a powder X-ray diffractometer (Bruker D8-Advance) covering an angular range of 2θ angle of 10^◦^–90^◦^ with a resolution of 2^◦^x10^− 2^. The Debye-Scherrer equation was applied to obtain the average particle size (D) Eq. (1).

1 $$\:D=\frac{K\gimel\:}{\beta\:\times\:Cos\:\theta\:}$$

Where D represents the average crystallite size measured perpendicular to the lattice planes. K is a numerical constant, often called the crystallite-shape factor, and typically has a value of around 0.9 (Wiley, 1974; Addison-Wesley, 1978). λ refers to the wavelength of the X-rays, specifically CuKa, which is 0.1542 nm. β is the full-width at half-maximum of the X-ray diffraction peak, expressed in radians, while θ denotes the Bragg angle, also in radians. FTIR was conducted to designate the functional groups. A mixture was prepared by combining 10 mg of AgNPs powder with 1 g of KBr powder to form Pellets for FTIR analysis. The FTIR spectrometer (Thermo Nicolet Nexus) was used and the analysis was conducted in the wavelength range of 4000–400 cm^− 1^ with 32 scans and a resolution of 1 cm^− 1^. To examine the external morphology of the synthesized AgNPs, a Field-Emission Scanning Electron Microscope (model FESEM, Zeiss SEM Ultra 60, 5 kV) was employed, along with an Energy Dispersive X-ray Analysis System (EDS) from Oxford. The samples were coated with gold prior to SEM examination. High-resolution transmission electron microscopy (Tecnai G2 Super Twin USA) was utilized to analyse the morphology and measure the particle diameters of the nanoparticles produced.

### Experimental fish

Two hundred apparently healthy Nile tilapia (*O. niloticus)* (average 29.5 ± 1.8 g in weight and 11.9 ± 1.6 cm in length) were collected from the farm of National Institute of Oceanography and Fisheries in Al-Qanater AL-Khayria, and transferred directly to the laboratory, fish transportation procedure was done conducted to^22^. The fish were acclimatized in 50-L aquaria filled with tap water (dechlorinated) and air was provided using air pumps under laboratory conditions for 14 days. All procedures were implemented in line with the directions specified in OECD (2023). Throughout the experiment period fish were fed a commercial pellet diet twice daily at 5% of their body weight. Water quality parameters were sustained during the experiment period as followed: 12:12 h light/dark photoperiod; water temperature 26.8 ± 1.3 ^◦^C; dissolved oxygen 6.8 ± 0.5 mg/ L; pH 7.1 ± 0.19; ammonia concentration 0.21 ± 0.06 mg/L using a multiparameter water quality meter (HORIBA U-52, Japan). The water was rehabilitated at a rate of 20% per day. The tank bottoms were siphoning on daily bases to remove fish feces and leftover meals. The experimental conditions were well-maintained during the acclimatization and experiment phase.

### Experimental design

The acclimatized Nile tilapia was divided into six groups, each consisting of 30 fish in triplicate. Over the course of six weeks, the first group served as a control, receiving no exposure to AgNPs or AgNO_3_. The second group (AgNO_3_) was exposed to silver nitrate (100 µg/L). The remaining groups (AgNPs-10, AgNPs-20, AgNPs-50, and AgNPs-100) were exposed to escalating doses of AgNPs (10, 20, 50, and 100 µg/L, respectively), following^23^.

### Fish sampling

All experimental procedures and methodologies of the current study were carried out in accordance with the Research Ethics Committee of the Faculty of Women for Arts, Ain Shams University (approval code: sci1332409001). Also aligned with the ARRIVE (Animal Research: Reporting of In Vivo Experiments) guidelines.

At the end of the experiment, fish were fasted for 24 h before being transferred from the holding tank and euthanised by immersing in an aesthetic solution containing clove oil (40 mg/L) for 5 min, following the method of^24^.

#### Liver samples collection and homogenation

Liver samples were collected from the experimental fish at three different time points, every two weeks, for the preparation of homogenates using a homogenizer (Omni TH-01). One gram of liver tissue was homogenized (10%, w/v) in ice-cold phosphate-buffered saline (0.1 M; pH 7.2). The homogenized tissues were then centrifugated at 15,000 rpm for 30 min at −4 °C. The resulting supernatant was preserved in liquid nitrogen for further analysis.

#### Hepatic antioxidant enzymes

The antioxidant parameters assessed include superoxide dismutase (SOD), catalase (CAT), glutathione peroxidase (GPx), and glutathione reductase (GR) activities. SOD activity was estimated according to the method of^25^, which measures the enzyme’s ability to inhibit the reduction of nitro blue tetrazolium dye at 560 nm, with results expressed as U/g tissue. CAT activity was measured using the^26^, which tracks the decomposition rate of hydrogen peroxide in the presence of catalase, with the reaction being halted by a catalase inhibitor at a specific time point. Readings were taken at 510 nm and stated as U/g protein.

GPx activity was assessed at 340 nm following the method of^27^, where the activity of GPx is inferred from the ability of glutathione reductase to oxidize NADPH to NADP^+^, with the reduction rate being directly related to GPx activity in the sample. Finally, the activity of GR was assessed by observing the reduction of glutathione disulfide (GSSG) in the presence of NADPH, which is converted to NADP + at a wavelength of 340 nm, as outlined by^28^.

#### Hepatic total antioxidant capacity

The assessment of total antioxidative capacity (TAC) involves the interaction of antioxidants in the sample using a known quantity of hydrogen peroxide (H₂O₂). The antioxidants neutralize a portion of the supplied H₂O₂, and the remaining H₂O₂ is quantified using a colorimetric enzymatic reaction with 3,5-dichloro-2-hydroxybenzenesulfonate at 505 nm^29^.

#### Hepatic oxidative stress biomarker

Lipid peroxidation (LPO), an indicator of oxidative stress, was estimated colorimetrically according to the thiobarbituric acid reactive substances (TBARS) method. This test measures the reaction between thiobarbituric acid (TBA) and malondialdehyde (MDA), with the absorbance of the resulting pink product recorded at 534 nm, as described by^30^.

### Histology

Immediately after dissection of the fish, liver specimens were fixed using formalin solution (10%). Subsequently, the specimens were sectioned and dehydrated using alcohol, then cleared with xylene, filtered in wax, and ultimately embedded in paraplast tissue embedding medium. A rotary microtome was employed to slice each sample into 5 μm thick sections. Following established procedures, the sections were stained with hematoxylin and eosin^31^.

### Statistical analysis

All data were presented as mean ± standard deviation (SD; *n* = 6 fish), statistical analysis was carried out using SPSS software (version 20; SPSS, USA). Kolmogorov examined the normality assumption of data–Smirnov. Tukey’s multiple range post-hoc test was applied following the one-way analysis of variance (ANOVA) was conducted to assess the significant differences between group means, with p-values less than 0.05 considered statistically significant.

## Results

### AgNPs characterization

**Fig. 1:**
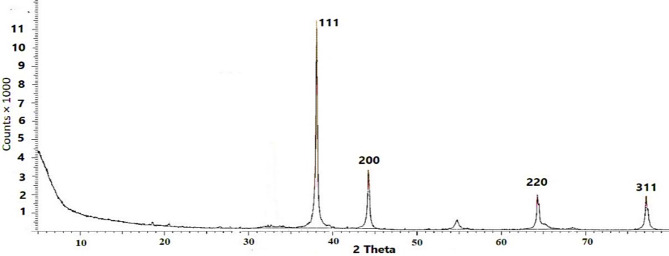
XRD of the synthesized AgNPs.

The XRD pattern provided evidence of the crystalline nature of AgNPs (Fig. 1). The four prominent diffraction peaks of 38.066, 44.224, 64.302, 77.291 can be indexed to the (111), (200), (220), and (311) reflection planes of the face-centred cubic structure of silver (JCPDS file No. 04-0783). The crystallite size ranged between from 27.44 to 35.02 nm with an average size of 29.92 nm, due to the Debye-Scherrer Equation.

**Fig. 2:**
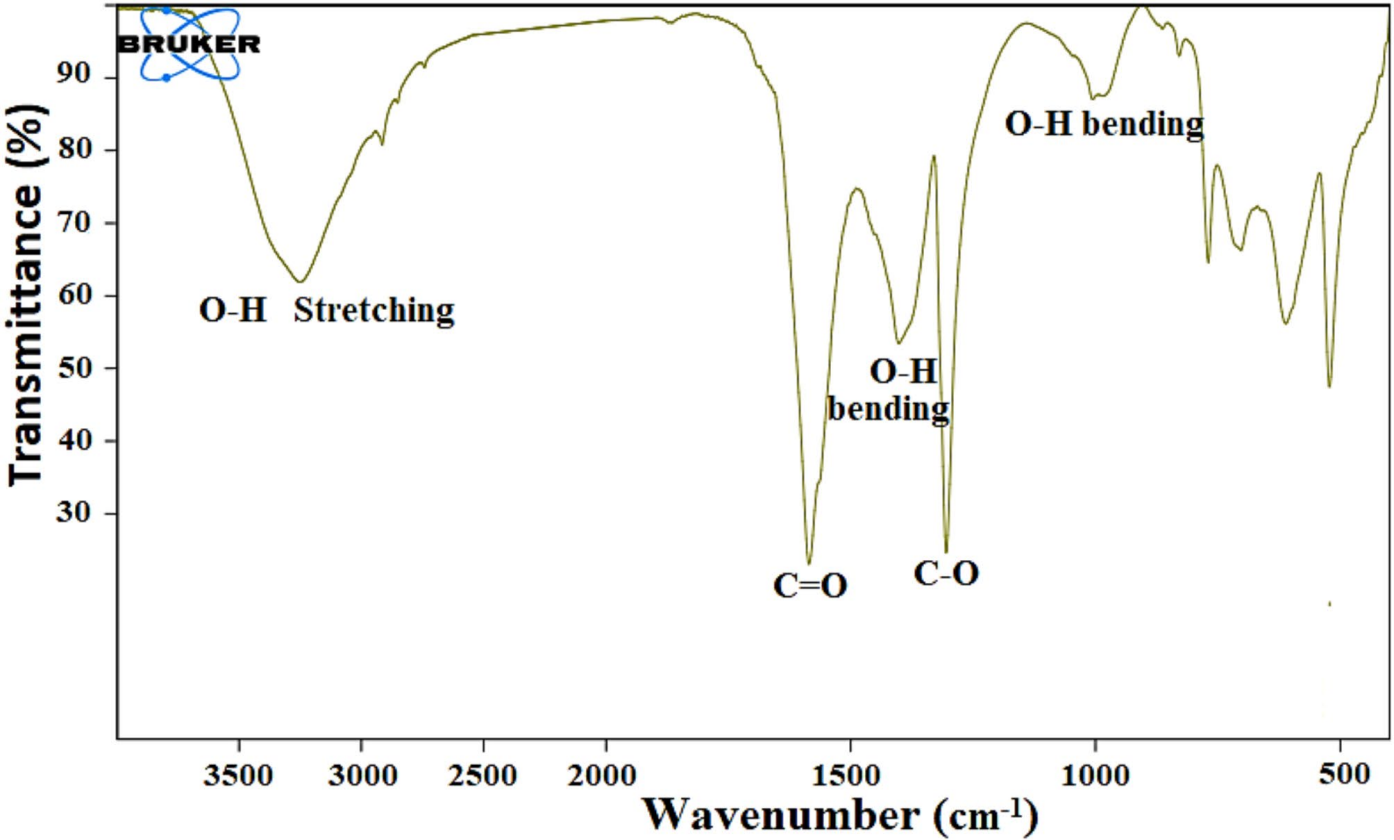
The FTIR of the synthesized AgNPs.

FTIR analysis is employed to explore and confirm the chemical composition of the materials^32^. A broad band that appears at 3250 cm^− 1^ may refer to the stretching vibration of the O-H group due to the adsorption of water on the surfaces of AgNPs, as it has a very high surface area. The bands at 1585.41 and 1304.81, may refer to the C=O and C–O stretching vibration on the AgNPs surface that results from the acetyl group in trisodium acetate employed as the stabilizing agent. The appearance of peaks at 1401.83 and 1005.6 may refer to the O–H bending vibrations, (Fig. 2). The portion of the infrared spectrum ranging from 1200 to 700 cm^− 1^ is referred to as the fingerprint region, which exhibits unique characteristics for every compound.

**Fig. 3:**
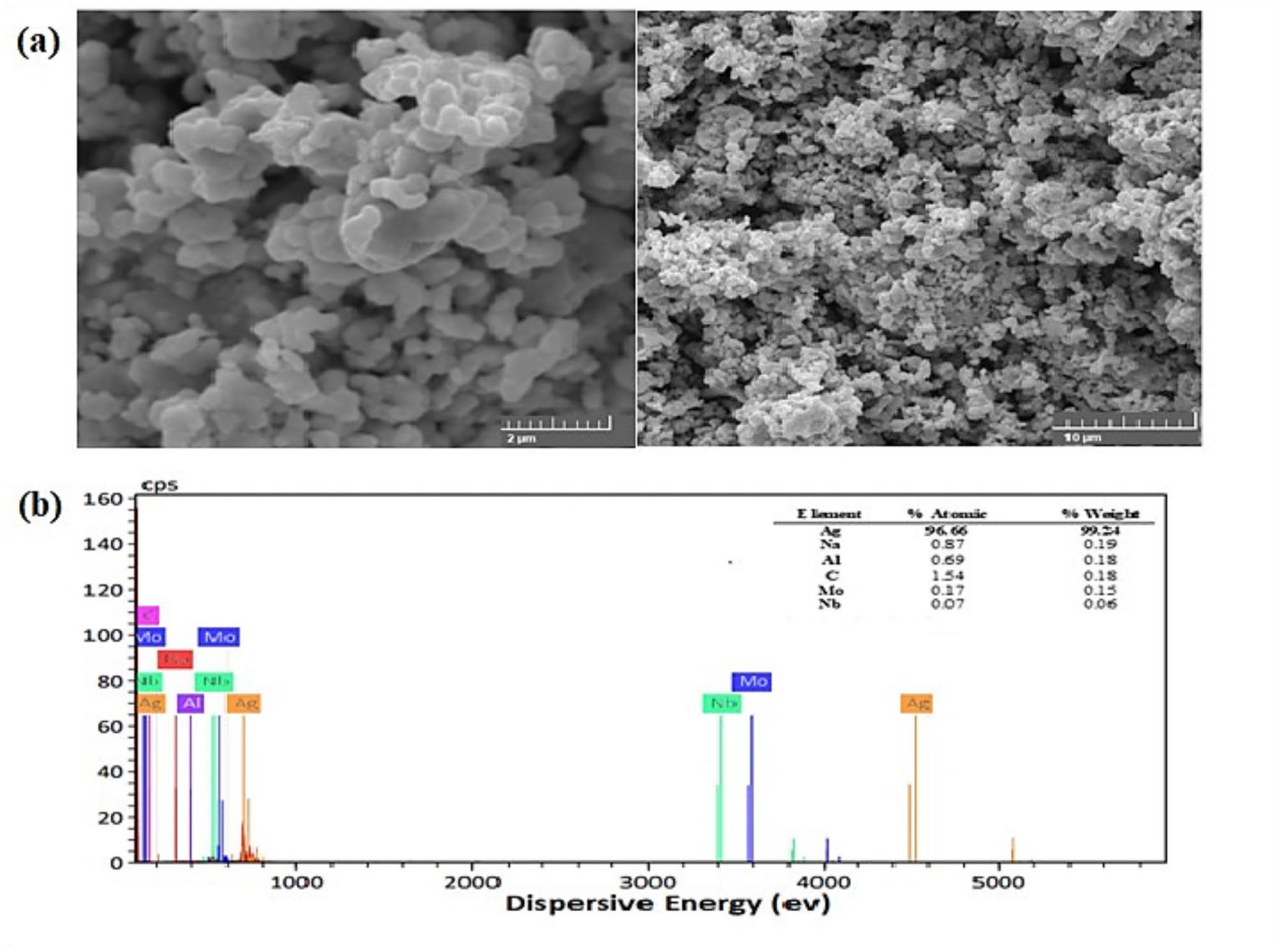
(**a**) SEM Images at different magnifications (2 and 10 μm) and (**b**) EDX elemental composition of the synthesized AgNPs.

The external morphology of the AgNPs was examined using SEM which confirmed the formation of AgNPs in a spherical shape with a highly porous surface. Figure 3a, b displayed the SEM images coupled with the elemental composition of the AgNPs. EDX showed the presence of metallic silver in 99.24% purity. Other interfering metals e.g., Na, Al, C, Mo and Nb exist in low percentages, 0.19, 0.18, 0.18, 0.15 and 0.06% (Fig. 3b).

**Fig. 4:**
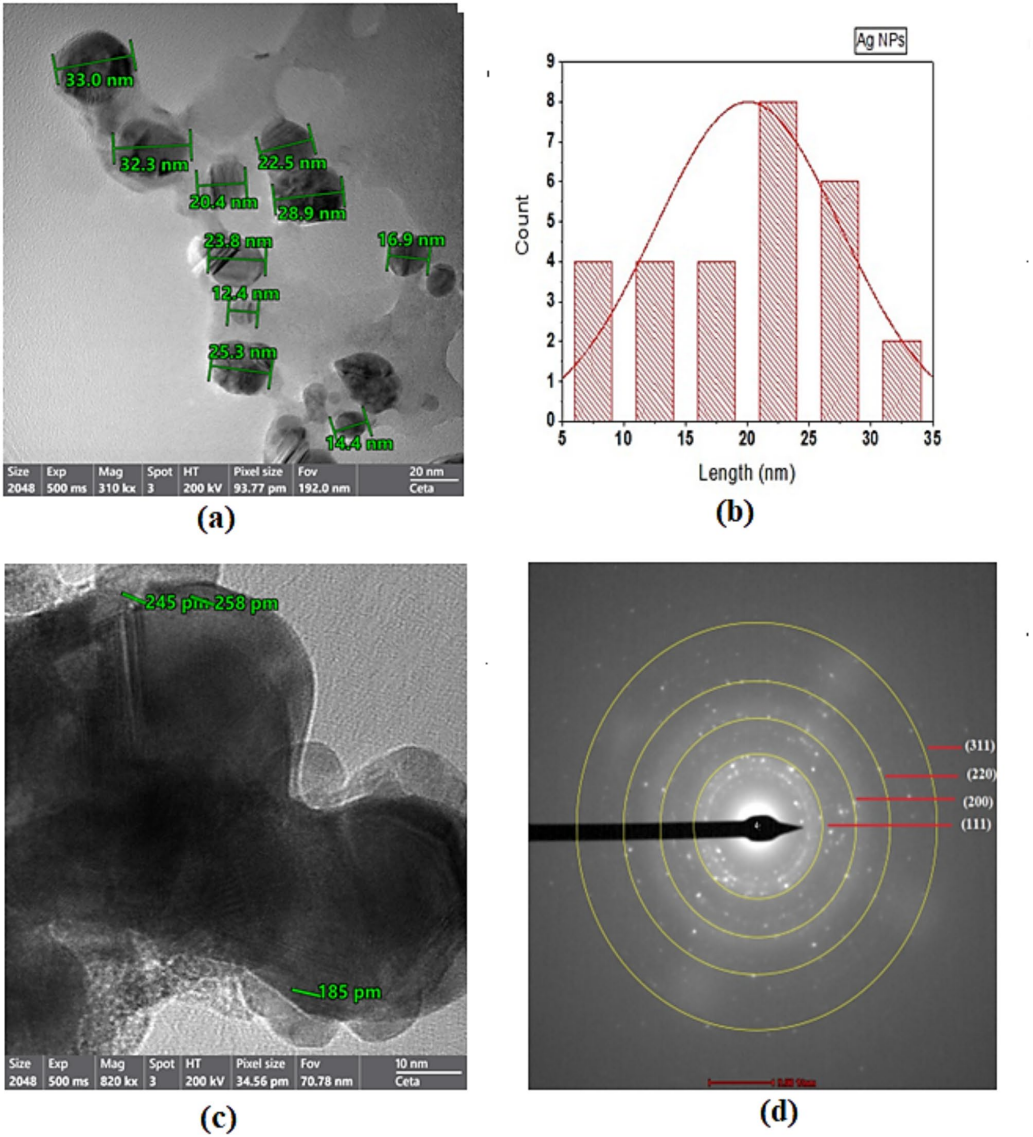
(**a**) TEM images of the synthesized AgNPs, (**b**) crystal size distribution, (**c**) HRTEM, (**d**) SAED pattern.

The TEM images (Fig. 4a) depict the production of AgNPs in a cubic-spherical morphology. The nanoparticles are arranged into clusters, forming numerous pores and cavities of high surface area and different biological activities. Moreover, it depicts the distribution of particle size between 12.4 and 33 nm, (Fig. 4b). HRTEM referred to an interplanar distance of 185 and 245 pm, (Fig. 4). When comparing the particle size determined using TEM with the size computed using the Debye-Scherrer equation using XRD, the results show a moderate level of similarity. Furthermore, selective area electron diffraction (SAED) refers to the polycrystalline pattern of the produced NPs with various diffraction rings ascribed to (111), (200), (220), and (311).

### Hepatic antioxidant defence system and LPO

Fish exposed to AgNo_3_ and AgNPs exhibited a noteworthy alteration in the activity of the antioxidant system and recorded an oxidative stress response during the experimental period. Compared with the control fish, AgNO_3_-exposed Fish recorded a significant elevation (*P* ≤ 0.05) by 1.1, 1.1, 1.2, 1.8, and 1.5-fold in SOD, CAT, GPx, GR and LPO activity in liver at week2, respectively (Table 1), these responses exhibited a continued upward trend during Week4 and week6. In week4, the activities of of SOD, CAT, GPx, GR and LPO demonstrated a significant increase, recording a 1.3, 1.1, 1.3, 2.5, and 1.9-fold elevation, whereas Week3 recorded a 1.4, 1.3, 1.3, 2.7, and 2 -fold increase in AgNO_3_-exposed fish compared to the control group.

**Table 1:** Hepatic antioxidant defense system and oxidative stress indicators (LPO) in Nile tilapia exposed to AgNO_3_ and various doses of AgNPs over a two-week period.

	Control	AgNo_3_	AgNPs 10	AgNPs 20	AgNPs 50	AgNPs 100
SOD (U/g)	51.8 ± 4.4^a^	61.7 ± 5.2^b^	58.3 ± 3.8^abc^	59.6 ± 4.6^abc^	62.2 ± 5.1^bc^	71.5 ± 4.3
CAT (U/g)	72.3 ± 3.7	83.4 ± 4.1^b^	76.3 ± 3.2^b^	79.8 ± 4.5^b^	92.4 ± 4.2^c^	98.2 ± 4.2^c^
GPx (U/g)	61.5 ± 3.4	77.6 ± 4.2^b^	71.1 ± 3.1^b^	77.9 ± 4.9^b^	81.2 ± 4.3^b^	90.7 ± 5.1
GR (U/g)	0.75 ± 0.3	1.32 ± 0.2^b^	1.07 ± 0.2^b^	1.23 ± 0.3^b^	1.93 ± 0.3^d^	2.16 ± 0.2^d^
LPO (nM/mg)	1.50 ± 0.2	2.31 ± 0.2^b^	2.13 ± 0.3^b^	2.30 ± 0.3^b^	2.56 ± 0.3^bc^	2.75 ± 0.2^c^
TAC (mM/mg)	3.57 ± 0.3	3.31 ± 0.2^b^	3.10 ± 0.5^bc^	3.13 ± 0.4 ^c^	2.81 ± 0.5^cd^	2.55 ± 0.3^d^

Data within a row with similar superscripts have no significant difference between them (Tukey Test, *P* < 0.05). Data were expressed as mean ± S.D. data (*n* = 6 fish).

*SOD* superoxide dismutase, *CAT* catalase, *GPx* glutathione peroxidase, *GR* glutathione reductase, *LPO* lipid peroxidase, *TAC* total antioxidant capacity.

TAC levels reported a remarkable decrease (*P* ≤ 0.05) by 1, 1.3, and 1.4-fold in liver of AgNO_3_-exposed fish during the study week, respectively (Table 2). Fish exposed to AgNPs demonstrated a dose-depended response in the antioxidant enzymes and LPO levels. Table 3 demonstrated a significant upsurge in SOD, CAT, GPx, and LPO levels in fish subjected to AgNPs at 10 and 20 µg/L during the experiment period, especially at week6 compared to control fish. TAC levels recorded a continuous substantial reduction (*P* ≤ 0.05) in AgNPs-10 and AgNPs-20 fish during the study period.

**Table 2:** Hepatic antioxidant defense system and oxidative stress indicators (LPO) in Nile tilapia exposed to AgNO_3_ and various doses of AgNPs over a four-week period.

	Control	AgNo_3_	AgNPs 10	AgNPs 20	AgNPs 50	AgNPs 100
SOD (U/g)	50.1 ± 4.2	63.4 ± 5.1^b^	61.8 ± 4.6^bc^	65.4 ± 4.2^bc^	67.8 ± 4.9^c^	74.1 ± 4.2
CAT (U/g)	74.2 ± 4.1	85.2 ± 4.3^b^	81.8 ± 3.8^b^	83.5 ± 3.4^b^	95.5 ± 4.9^c^	102.5 ± 6.7^c^
GPx (U/g)	59.2 ± 3.8	78.1 ± 2.7	73.4 ± 3.6^b^	69.8 ± 3.1^b^	63.7 ± 2.7^b^	68.9 ± 3.8^b^
GR (U/g)	0.76 ± 0.2^a^	1.93 ± 0.2	1.28 ± 0.1^b^	1.32 ± 0.2^b^	1.15 ± 0.2^b^	0.72 ± 0.3^a^
LPO (nM/mg)	1.38 ± 0.2	2.66 ± 0.3^b^	2.31 ± 0.2^bc^	2.41 ± 0.3^bc^	3.34 ± 0.3^d^	3.26 ± 0.2^d^
TAC (mM/mg)	3.46 ± 0.3	2.61 ± 0.1^b^	2.51 ± 0.2^bc^	2.33 ± 0.2^bcd^	2.71 ± 0.2b^c^	2.32 ± 0.1^bd^

Data within a row with similar superscripts have no significant difference between them (Tukey Test, *P* < 0.05). Data were expressed as mean ± S.D. data (*n* = 6 fish).

*SOD* superoxide dismutase, *CAT* catalase, *GPx* glutathione peroxidase, *GR* glutathione reductase, *LPO* lipid peroxidase, *TAC* total antioxidant capacity.

**Table 3:** Hepatic antioxidant defense system and oxidative stress indicators (LPO) in Nile tilapia exposed to AgNO_3_ and various doses of AgNPs over a six-week period.

	Control	AgNo_3_	AgNPs 10	AgNPs 20	AgNPs 50	AgNPs 100
SOD (U/g)	52.4 ± 3.8	72.1 ± 3.1^b^	63.1 ± 3.0^c^	66.7 ± 3.3^bc^	48.6 ± 2.7^d^	44.2 ± 2.5^d^
CAT (U/g)	72.9 ± 3.1	94.3 ± 3.0^b^	87.4 ± 2.7^c^	91.1 ± 2.4^bc^	64.3 ± 2.3^d^	60.1 ± 2.7^d^
GPx (U/g)	59.2 ± 3.8	79.2 ± 4.2^b^	76.2 ± 3.1	80.8 ± 3.7^b^	51.1 ± 4.3^d^	45.8 ± 4.8^d^
GR (U/g)	0.79 ± 0.3	2.17 ± 0.1	1.47 ± 0.3^c^	1.57 ± 0.2^c^	0.61 ± 0.1^d^	0.56 ± 0.1^d^
LPO (nM/mg)	1.52 ± 0.3	3.04 ± 0.3^b^	2.97 ± 0.3^bc^	2.92 ± 0.2b^c^	3.73 ± 0.3^d^	4.21 ± 0.4^d^
TAC (mM/mg)	3.53 ± 0.4	2.43 ± 0.5^b^	2.23 ± 0.4^bc^	2.16 ± 0.3^bcd^	1.86 ± 0.5^bcd^	1.67 ± 0.5^bcd^

Data within a row with similar superscripts have no significant difference between them (Tukey Test, *P* < 0.05). Data were expressed as mean ± S.D. data (*n* = 6 fish).

*SOD* superoxide dismutase, *CAT* catalase, *GPx* glutathione peroxidase, *GR* glutathione reductase, *LPO* lipid peroxidase, *TAC* total antioxidant capacity.

The effect of AgNPs was demonstrated significantly at higher doses (AgNPs-50 and AgNPs-100), week2 recorded a remarkable increase (*P* ≤ 0.05) in SOD, CAT, GPx, GR, and LPO levels by 1.2, 1.3, 1.3, 2.6, and 1.7-fold in AgNPs50-exposed fish, and by 1.4, 1.4, 1.5, 2.9, and 1.8-fold in AgNPs100 fish, compared to control fish (Table 1), on the other hand, TAC levels decreased significantly (*P* ≤ 0.05) in AgNPs-50 and AgNPs-100 fish by 1.3 and 1.4-fold compared to control fish, individually.

Week4 recorded a significant increase (*P* ≤ 0.05) in antioxidant enzymes activity and LPO levels in fish subjected to high AgNPs levels, while GR levels exhibited non-significant elevation in AgNP-100 fish, relative to control group. TAC levels were significantly reduced in fish exposed to high AgNPs levels, as AgNPs-50 and AgNPs-100 fish reported 1.3 and 1.5-fold lower TAC levels than control fish, as shown in (Table 2).

Week6 recorded a significant inhibition (*P* ≤ 0.05) in SOD, CAT, GPx, GR, and TAC levels by 1, 1.1, 1.2, 1.3, and 1.9-fold in AgNPs-50 fish and by 1.2, 1.2, 1.3, 1.3, and 2.1-fold in AgNPs-100 fish, respectively in relation to the control fish. LPO levels exhibited a substantial elevation (*P* ≤ 0.05) in both AgNPs-50 and AgNPs-100 groups (2.4 and 2.8-folds) compared to control group (Table 3).

### Histopathological investigation

**Fig. 5:**
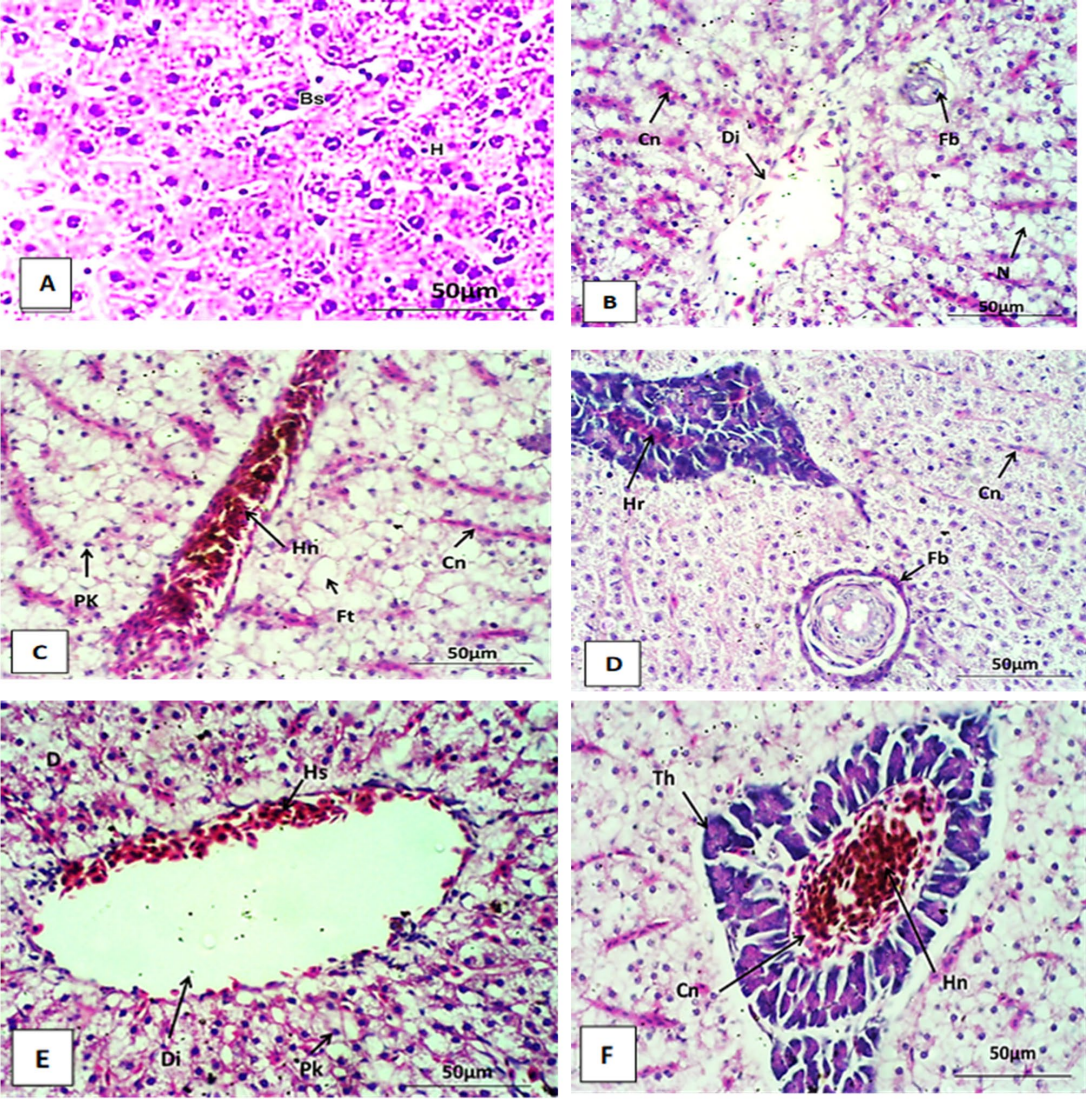
liver section of *Oreochromis niloticus* after two weeks stained with Hx&E at 400X. (**A**) liver section of control showing normal hepatocytes and normal blood sinusoid. (**B**) liver section exposure to AgNO_3_ (100µ/L) showing congestion (Cn) and dilatation (Di) of blood vessel, necrosis (N) and fibrosis (Fb) in hepatocytes. Respectively, (**C**–**F**) showing histological alteration in liver sections after exposure to AgNPs with concentrations (10, 20, 50 and 100 µ/L). These alterations include hemorrhage (Hr) (**D**), Congestion (cn) (**C**,**D**,**F**), fatty degeneration (Ft) (**C**), fibrosis (Fb) (**D**), hemosiderin pigments (Hs) (**C**,**F**), dilation of blood vessels (Di) (**E**), thickness of blood vessel (Th) (**F**) and pyknosis of nuclei (Pk) (**C**,**F**).

**Fig. 6:**
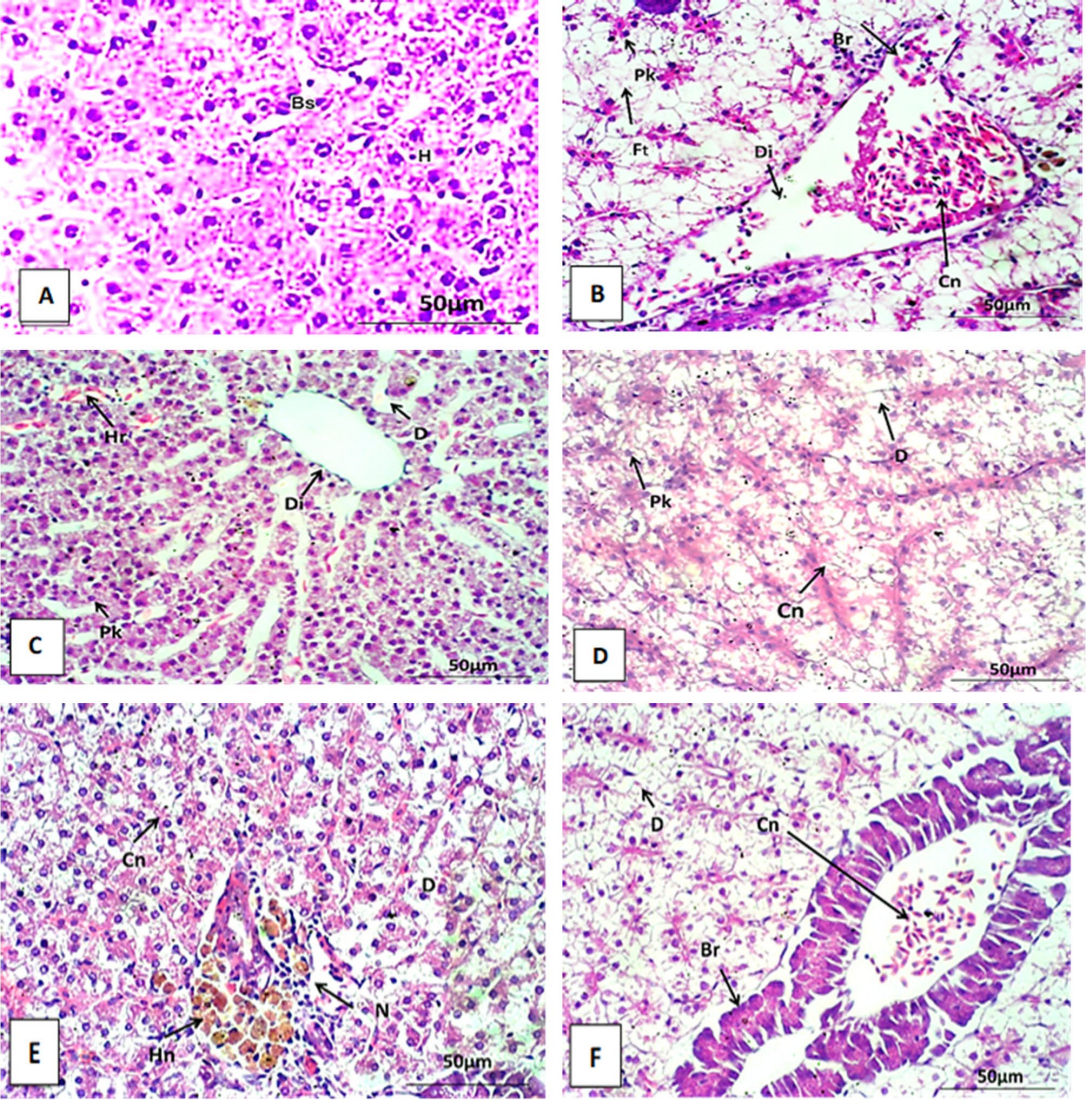
liver section of *Oreochromis niloticus* after 4weeks stained with Hx&E at 400X. (**A**) liver section of control showing normal hepatocytes and normal blood sinusoid. (**B**) liver section exposure to AgNO_3_ (100µ/L) showing congestion (Cn), dilatation (Di) and branching (Br) of blood vessel, fatty degeneration (Ft) and pyknosis of nuclei (Pk). Respectively, (**C**–**F**) showing histological alteration in liver sections after exposure to AgNPs with concentrations (10, 20,50 and 100 µg/L). The histological alterations include hemorrhage (Hr) (Fig. 8), Congestion (cn) (**D**–**F**), fatty degeneration (Ft) (Fig. 3), hemosiderin pigments (Hs) (**E**), dilation of central vein (Di) (**C**) and branching of blood vessel (Br) (**F**), pyknosis of nuclei (Pk) (**D**), necrosis (N) and degeneration (D) between hepatocyte (**C**–**F**).

**Fig. 7:**
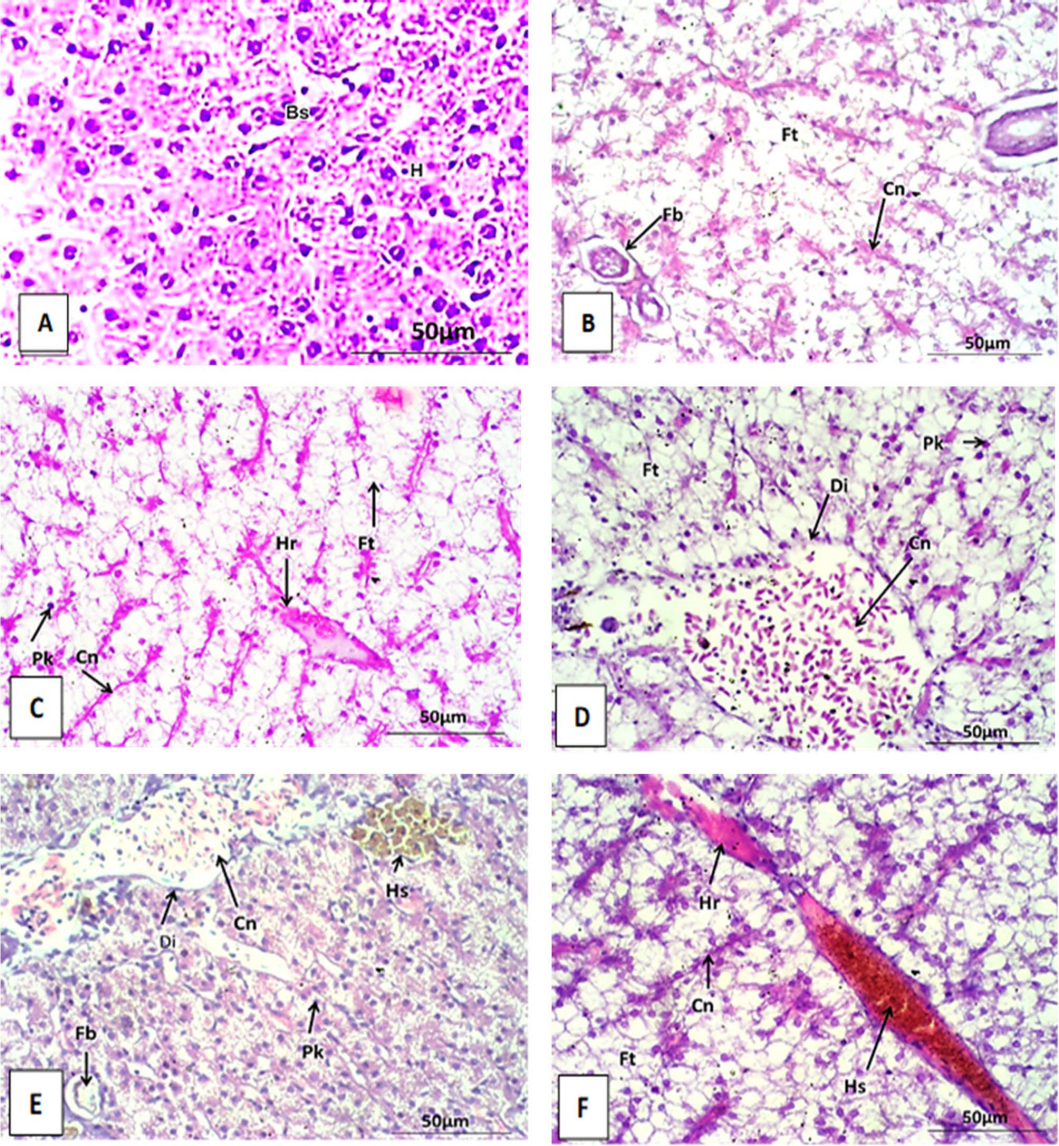
liver section of Oreochromis niloticus after 30 days stained with H&E at 400X. (**A**) liver section of control showing normal hepatocytes and normal blood sinusoid. (**B**) liver section exposure to AgNO_3_ (100µ/L) showing congestion (Cn), fatty degeneration (Ft) and fibrosis (Fb) between hepatocytes. Respectively, (**C**–**F**) showing histological alteration in liver sections after exposure to AgNPs with concentrations (10, 20, 50 and 100 µ/L). The histological alterations include hemorrhage (Hr) (**C**,**F**), hemolysis (Hs) (**F**), Congestion (cn) (**B**–**F**), fatty degeneration (Ft) (**C**,**D**,**F**), hemosiderin pigments (Hs) (**E**), dilation of blood vessels (Di) (**D**,**E**), pyknosis of nuclei (Pk) (**C**–**E**) and necrosis (N) between hepatocyte (**F**).

The liver of the control Nile tilapia is composed of hepatocytes lined in a distinct cord-like pattern around definite sinusoids around a central vein. hepatocytes are multilateral with centrally located nuclei. Blood streams from the hepatic portal vein branches and artery through the sinusoids to the main central veins, then drain into the hepatic vein (Figs. 5A and 6A, and Fig. 7A).

The histological investigation of Nile tilapia liver tissues, conducted every two weeks over a six-week period of AgNPs exposure, revealed significant pathological alterations. By weeks 2, 4, and 6, the observed changes included congestion, dilatation, and branching of blood vessels (see Figs. 5B and 6B). These findings indicate progressive liver damage associated with AgNPs exposure. Additionally, fibrosis, fatty degeneration, and necrosis were detected in the hepatocytes (see Figs. 5B and 6D, and Fig. 7B). The extent of these histopathological lesions increased with the duration of exposure to AgNO_3_. Similarly, fish exposed to AgNPs at concentrations of 10, 20, 50, and 100 µg/L showed a range of histopathological alterations. These changes became progressively more severe with both longer exposure and higher concentrations of AgNPs, indicating a dose- and time-dependent exacerbation of liver damage.

Over the course of six weeks, several pathological changes were observed in the liver, including dilatation of the central vein and blood vessels (Fig. 5E), as well as congestion and thickening of the vessel walls (see Figs. 5C and F and 6E and F, and Fig. 7E). Hemorrhage between hepatocytes, hemosiderin pigment deposition, and fatty degeneration were also noted (see Figs. 5F, 6C and 7C and F). Additional alterations included fibrosis, pyknotic nuclei, and necrosis within hepatocytes (see Figs. 5D and 6E, and Fig. 7D).

## Discussion

Nanotechnology is a growing emerging technique in which many scientists are concerned due to its potential applications, especially in aquaculture^33^. This study investigates the potential relative toxic effects of AgNO_3_ and synthesized AgNPs on oxidative stress, antioxidant enzymatic activities, and histological architecture of the liver of freshwater fish (Nile tilapia). The findings of this study demonstrated that both antioxidant enzyme activities and lipid peroxidation levels in Nile tilapia exhibited a dose-dependent response.

### Synthesized AgNPs

AgNPs are a unique type of silver with a size of less than 100 nm. This small size allows for a high ratio of surface area to volume that exposes different biological activities^34^. The XRD pattern provided evidence of the crystalline nature of AgNPs and referred to a face-centered cubic structure with a crystallite size (< 50 nm) ranging from 27.44 to 35.02 nm. FTIR demonstrates the functional groups on the AgNPs surface; –OH, C=O, C–O. These groups resulted from acetyl groups responsible for the stability of the AgNPs during the synthesis. SEM examined the external morphology of the AgNPs and confirmed its formation in a spherical shape with a highly porous surface.

This shape is the most popular one for AgNPs^35^. EDX confirmed the high purity of AgNPs by the presence of metallic silver in 99.24% purity. HRTEM verified the full reduction of all silver ions in the nanoscale (100 nm) with a high level of stability of the generated nanoparticles within a limited particle size range (12.4–33 nm). Different reducing and stabilizing agents were employed in previous works for the AgNPs synthesis such as; glucose, ascorbic acid, oxalic acid, NaBH_4,_ and trisodium acetate. However, this study pertained to the excellent efficacy of trisodium acetate in reducing Ag ions and stabilizing the synthesized NPs. The crystallite size calculated from the Debye Scherrer Equation was somewhat close to that evaluated from The TEM Images. Furthermore, SAED referred to the polycrystalline pattern of the produced NPs and confirmed the reflection planes of XRD results.

### Hepatic antioxidant enzymes and LPO

The antioxidant system is designed to protect the cell from the damage of ROS, Superoxide dismutase (SOD), catalase (CAT), and glutathione peroxidase (GPx) are recognized as key antioxidant enzymes responsible for mitigating cellular oxidative damage. They primarily function is to neutralize or inhibit the formation of free radicals within the cell. SOD is considered the most powerful antioxidant enzyme, initiating the detoxification process by converting superoxide anions into hydrogen peroxide and molecular oxygen. This process mitigates the potential harm of superoxide anions. Then, CAT further reduces hydrogen peroxide into water and oxygen, efficiently managing the by-products of SOD’s activity. CAT operates with remarkable efficiency, neutralizing millions of hydrogen peroxide molecules per second. GPx complements this by converting hydrogen peroxide into water and transforming lipid peroxides into alcohols, thereby playing a crucial role in mitigating oxidative stress and limiting lipid peroxidation (LPO)^36^. The physiological responses related to stress are crucial for preserving homeostasis and safeguarding the health of fish. While stress induces adaptive responses to difficult environmental conditions, it can also have detrimental effects on fish^37^.

In addition to these enzymatic antioxidants, the ascorbate-glutathione cycle’s primary non-enzymatic antioxidants, particularly those involving glutathione, are critical. Since cellular redox reactions depend on enzyme-mediated catalysis for proper specificity and reaction rates, glutathione-related enzymes are of significant interest. Glutathione reductase (GR), an NADPH-dependent enzyme, helps the reduction of oxidized glutathione (GSSG) to its reduced form (GSH)^36^.

The liver is known as a critical site for enzyme activity that enable the oxidative degradation of toxins such as metals, stimulating the generation of reactive oxygen species (ROS). This leads to enhanced LPO levels, as detected in our study. In aerobic organisms, phospholipid membranes in vital organs, are regularly imperilled to internal and external sources oxidative stress^38^. Our results confirms that the liver is the most important tissue for studying oxidative stress in fish due to its sensitivity to toxins and profound role in detoxification and biotransformation of contaminants. Basically, water contaminants induce various stress responses, that primarily persuades oxidative stress markers in Nile tilapia such as high levels of LPO^39–41^. Both AgNO_3_ and AgNPs levels used in the present study were environmentally relevant as AgNO_3_ and AgNPs were detected in different water bodies^42^.

Silver naturally may exist in different forms, however ionic silver is extremely harmful^43^. Bulk and nano-silver is related to damage of vital constituents in the cell including; protein, DNA, and lipid. Free radical over production can suppress the antioxidant defense system, which encourage further cellular oxidative damage, displayed in higher LPO levels due to the oxidative degradation of the fatty acids^44^.

The current study observed an activation of the antioxidant system’s capacity and an increase in oxidative stress biomarkers at week 2 and 4, indicating significant cellular damages of fish exposed to both forms of silver, especially AgNPs. This oxidative damage was revealed by higher levels of SOD, CAT, GPx, GR, and LPO and lower TAC levels in the liver of Nile tilapia exposed to AgNo_3_ and AgNPs at different levels for 2 and 4 weeks. While the chronic exposure to AgNPs, especially in higher dose (AgNPs-100), displayed further oxidative stress responses in week6, as all antioxidant enzymes were suppressed and the LPO levels were remarkably higher, compared to the control and corresponding AgNO_3_ group. In coherence with the attained results, previous studies reported similar SOD, CAT, and GPx enzymatic activation due to toxic metals’ exposure^40,45^. Metals can interrupt the cellular redox equilibrium and boost radical production, such as superoxide radicals, which may surpass the Competence of antioxidant enzymes to handle^46^. Assembly of oxygen radicals, leading to oxidative stress, which can harm various cellular constituents. The antioxidant system serves as a defending network versus oxidative injury from radicals, using several mechanisms such as neutralizing radicals, controlling radical production, and restoring radical-induced injury. Although, radicals play pivotal roles in the immune system, cellular signalling, and sustaining redox equilibrium, overwhelming radicals such a ROS can denaturates lipids, proteins, DNA, and subcellular units, possibly activating apoptosis^47^.

LPO is a typical product of ROS-induced damage, where radicals attack the polyunsaturated fatty acids, starting a cascading effect. Oxidative stress indicates a redox imbalance Identifiable by the Overabundance of ROS and the following peroxidation of proteins, phospholipids, and DNA^48^. In week 2 and 4, the oxidative damage caused by AgNO_3_ was greater than that caused by AgNPs-10 and AgNPs-20, whereas higher doses of AgNPs resulted in increased LPO and decreased TAC compared to AgNO_3_. This suggests that while AgNO_3_ may initially cause more oxidative stress, leading to the activation of the antioxidant system, higher concentrations of AgNPs result in more significant LPO and a reduction in TAC levels. This potentially indicates severe impacts on the oxidative balance within the fish.

In week6, the exposure of AgNPs-100 displayed a descending trend in hepatic antioxidant-related enzymes in Nile tilapia along with higher LPO levels, signifying that the accretion of ROS was enough to overwhelm the antioxidant path enzymes and their functions. A dose–dependent manner was revealed in the present study, with alterations in antioxidant enzyme activities and LPO levels of Nile tilapia in response to different concentrations of AgNPs. This advocates that the antioxidant defense system of fish exposed to AgNPs-100 for six weeks breakdowns and drops its capacity to moderate cellular oxidative damage^8^. Although antioxidant enzyme activities were enhanced in the AgNO_3_, AgNPs-10 and −50 groups related to the control fish, this enhancement could be a coping strategy to mitigate the oxidative stress induced by silver metal^49^. This finding is consistent with^50^, who documented higher SOD, CAT, and GPx levels in fish exposed to different doses of the AgNPs (0–0.5 mg L^− 1^). Moreover, LPO levels specify cellular injury in fish caused by AgNO_3_ and AgNPs-induced oxidative stress^16,8^.

Ref^51^. reported that the accumulations of silver in zebrafish were significantly correlated with those of AgNPs, compared to fish exposed to AgNO_3_, concluding that the increased toxicity of AgNPs relative to silver may be attributed to their size and/or shape. AgNPs have recently received growing interest owing to their high toxicity to aquatic animals, including *C. carpio*^52^. *O. mossambicus*^8^; zebra fish^53^; *P. mesopotamicus*^54^; *Cyprinus carpio*^55^. Eurasian perch exposed to high dose of AgNO_3_ (386 µg/L) reported a substantial hypoxia, while the exposure to both AgNO_3_ and AgNPs reduced the fish capacity to mitigate the induced hypoxia^52^.

Ref^56^. reported a decline in the activity of CAT as a supportive report for the achieved results owing to metal binding, which affect the enzyme activity. AgNPs doses persuades a similar reduction in antioxidant enzymes activity, but to a larger extent than exposure to AgNO_3_ in chronic exposure (week6). As outlined in the report by^57^, the activity of glutathione-related enzymes (GPx and GR) in the liver connected to the enzymatic responses to toxic nanoparticles, playing a key role in removing pollutants and harmful substances to protect the cells. In support of this, the current study displayed a reduction in GST and GR activities in prolonged exposure to higher doses of AgNPs (AgNPs-50 and − 100), while LPO levels increased. Generally, GST and GR levels show species- and tissue-specific alterations in many fish after metal exposure^22,37,47,48,54,58–61^. However, the mechanisms underlying AgNPs toxicity are not fully understood and result in complex oxidative stress^1^.

Our findings support the previously mentioned hypotheses that the chronic exposure of high AgNPs doses can reinforce the production of ROS and decrease the glutathione-related enzymes, consequently lower the capacity of the antioxidant system to overcome the induced cellular damage. The observed suppression of the fish’s antioxidant defense enzymes due to AgNPs exposure at week 6, particularly at higher doses, may be related to the noxious impacts of AgNPs, which induce physiological stress due to the leakage of silver ions into the fish’s system. This release has been reported to obstruct sulfhydryl agents within mitochondria, disrupting the redox balance within cells by impairing the scavenging of reactive oxygen species (ROS)^62^. Similar findings were stated by^63^ who reported a noteworthy upsurge in both ROS production and reduction of the activity of antioxidant enzyme related to the reference group. Moreover, fish exposed to AgNPs showed an increase in oxidative phosphorylation and a disruption in protein synthesis^64^. Consequently, the current findings might suggest that the elevated LPO levels, significantly linked to the induction of oxidative stress through excessive radical production, as reported by^59^.

Yellow Perch displayed similar inhibition of both CAT and GPx expressions after exposure to AgNPs^65^. Conversely, high doses of AgNPs have been attributed to GPx gene inhibition together with its activity^60^. Additionally, AgNPs contact persuaded an upregulation of genes involved in protein denaturation and led to the downregulation of growth hormone^52^.

The redox status of Nile tilapia exposed to AgNPs displayed an activation of SOD, CAT, GPx, and GR enzyme activities. However, as the exposure period increased, there was a noticeable decline in the antioxidant enzymes’ activities. The initial activation of these antioxidant enzymes at beginning of the exposure may be attributed to the compensatory response of the antioxidant to mitigate the toxic effects of AgNPs. Similarly^66^, recorded higher antioxidant enzyme activities in different tissues of Indian major carp (*Labeo rohita*) when exposed to AgNPs (100 µg/kg), likely due to the activation of defensive mechanisms against oxidative stress.

### Histopathological findings

Histological alterations, as studied previously and confirmed by the present study, have proven to be valuable for evaluating the harm triggered by AgNO_3_ and nanomaterials^67^. The liver, a critical organ responsible for active metabolism and detoxification, is particularly sensitive to pollutants^40^. The current results indicate that AgNPs induce varying degrees of pathological changes in liver tissue. These findings are consistent with observations in *O. mossambicus* subjected to nanoparticles of nickel^68^ and in *Labeo rohita* followed the exposure to AgNPs^66^. Similar histopathological changes in the liver have also been documented by^69–71^.

## Conclusion

The findings of this study demonstrated that both antioxidant enzyme activities and lipid peroxidation levels in Nile tilapia exhibited a dose-dependent response. Underscore the destructive consequences of both bulk silver nitrate and silver nanoparticles on Nile tilapia, with a more pronounced impact observed with silver nanoparticles exposure for longer period. The increased oxidative stress, as indicated by elevated antioxidant enzyme activities and lipid peroxidation, coupled with a significant reduction in total antioxidant capacity, demonstrates a mitigation response to the pronounced toxicity of silver bulk and nanoparticles. Histopathological analysis further revealed extensive liver damage, including congestion, fibrosis, and necrosis, which escalated with higher nanoparticle concentrations and extended exposure periods. These results suggest that silver nanoparticles pose a greater environmental and physiological risk compared to bulk silver, highlighting the need for cautious use and regulation of nanomaterials in aquatic environments. Future studies should focus on uncovering the mechanisms of nanoparticle toxicity and developing approaches to mitigate their consequences on aquatic organisms, with a focus on potential recovery mechanisms after exposure ends.

## Data Availability

The data supporting the findings of this study are available within the article.
